# Associative memory cells: Formation, function and perspective

**DOI:** 10.12688/f1000research.11096.2

**Published:** 2017-03-30

**Authors:** Jin-Hui Wang, Shan Cui

**Affiliations:** 1 Institute of Biophysics, Chinese Academy of Sciences, Beijing, 100101, China; 2 College of Life Sciences, Chinese Academy of Sciences, Beijing, 100101, China; 3 School of Pharmacy, Qingdao University, Qingdao, 266021, China

**Keywords:** Associative memory cell (AMC), synapse, neuron, learning and cognition

## Abstract

Associative learning and memory are common activities in life, and their cellular infrastructures constitute the basis of cognitive processes. Although neuronal plasticity emerges after memory formation, basic units and their working principles for the storage and retrieval of associated signals remain to be revealed. Current reports indicate that associative memory cells, through their mutual synapse innervations among the co-activated sensory cortices, are recruited to fulfill the integration, storage and retrieval of multiple associated signals, and serve associative thinking and logical reasoning. In this review, we aim to summarize associative memory cells in their formation, features and functional impacts.

Associative learning is a common approach for information acquisition, and associative memory is essential for logical reasoning, associative thinking, comparison and computation ^1– 4^. Each object possesses several characteristics that can be detected by different sensory modalities. An apple is detected by the olfactory system for its perfume, the visual system for its shape and color, the taste system for its sweetness, the auditory system for its name, and so on. In initial associative learning, how do the sensory cortices integrate these cross-modal signals for us to describe an object and fulfill their associative memory? i.e., how does the brain jointly store multiple signals and distinguishably retrieve them? In fact, such signals can be retrieved reciprocally, i.e., one signal induces the recall of its associated signals, or the other way around. The mutual synapse innervations among the co-activated sensory cortices and the associative memory cells in these areas are presumably recruited during associative learning ^1, 3, 5– 7^.

In the studies of cellular and molecular mechanisms underlying associative learning and memory, animal models in fear conditioning, eyelid-blinking conditioning and operant conditioning are used ^8– 11^. A psychological view suggests that a conditioned signal induces the prediction of an unconditioned forthcoming signal as the basis of conditioned reflexes; however, the cellular mechanisms remain unclear ^4^. Activity-dependent neural plasticity, such as long-term potentiation ^12^ and depression ^13^, is presumably involved. Whether these types of neural plasticity are correlated with these associated signals remains to be examined. In addition, perceptual memory presumably resides in the cell assembles formed by the strengthening of neuronal connections due to their correlated activities during information acquisition ^14^. However, the nature of these memory cells is largely unknown.

A few of points are worth considering in the use of these conditioning models. Associative memory appears as a signal evoking the recall of its associated signals, or the other way around. After fear conditioning or eye-blinking conditioning is established, whether the air-puffing to the cornea or the electrical shock to the feet induces the recall of the sound signal remains unknown. Moreover, the electrical shock may activate entire sensory cortices and even the whole brain through the spread of electrical currents to all the sensory systems in the body, so that the association is not region-specific in the brain. In addition, the cerebellum may not be a primary region for the joint storage of air-puff to the cornea and sound.

## A comprehensive model of associative memory

Current reports show the reciprocal form of cross-modal reflexes in mice, i.e., paired odor and whisker stimulations lead to odorant-induced whisker motion and whisker-induced olfaction responses ^5, 6^. This mutual retrieval of associated signals can be used to explain associative memory. After two signals are learnt associatively, one signal induces the recall of its associated signals by the presentation of their respective behaviors, or the other way around, so that individuals are able to fulfill logical reasoning and associative thinking in forward and backward manners. For instance, people looking at an orange recall its sour-sweetness to be salivary, and people tasting something that has sour-sweetness may recall an orange or orange-like juice.

Where is the location to integrate the associated signals? Since inhibiting the function of sensory cortices blocks the reciprocal cross-modal reflex ^1, 3, 15^, the primary area for associative memory is likely located in sensory cortices, where mutual synapse innervations and associative memory cells are recruited after associative learning ^1, 3, 7^.

## The cellular mechanism underlying associative memory

The associations of sensory signals lead to their associated storage and retrieval, so that each signal is able to induce the recall of other signals. Cellular substrates are hypothetically based on the events that the co-activations of the sensory cortices, by pairing their input signals, recruit the mutual synapse innervations among these cortices for integrating these associated signals and the associative memory cells for encoding these signals in sensory cortices ^5, 6^.

After odorant-induced whisker motion and whisker-induced olfaction responses are established in mice, their barrel and piriform cortical neurons are recruited to encode a new signal alongside an innate signal. Barrel cortical neurons encode new odor and innate whisker signals. Piriform cortical neurons are able to encode new whisker and innate odor signals. Moreover, barrel cortical neurons receive new synapse innervation from the piriform cortex alongside an innate one from the thalamus. In addition, piriform cortical neurons receive new synapse innervations from the barrel cortex alongside innate ones from the olfactory bulb. That is, barrel and piriform cortical neurons are mutually innervated through their axons and synapse outputs ^1, 3, 5, 6^. The neurons that encode both new and innate signals based on their mutual synapse innervations are named as associative memory cells. The neurons that encode either one of signals are called new memory cells or innate memory cells. Associative memory cells include glutamatergic neurons, GABAergic neurons and astrocytes ^1, 3, 7^. miRNA-mediated epigenetic processes also appear to be involved ^7, 15^. Associative memory cells and mutual synapse innervations among sensory cortices constitute the cellular substrates for memory to specific associated signals. Notably, associative memory cells are able to store more than two signals ^15, 16^. For instance, paired whisker, odor and tail stimulations lead to odorant-induced and tail-induced whisker motions alongside whisker-induced whisker motion. The neurons in these sensory cortices are recruited to encode these three signals through mutual cortical innervations ^15^.

**Figure 1.**
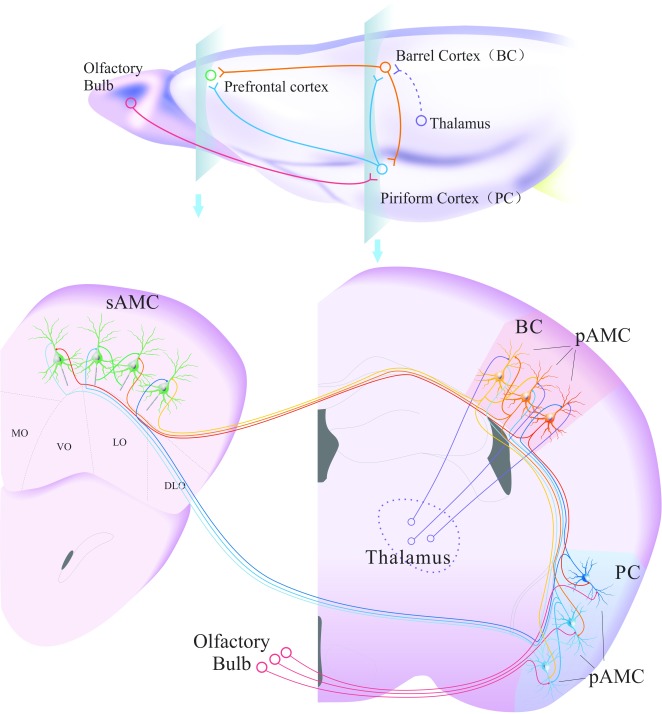
The associated activation of the sensory cortices recruits their mutual innervations and associative memory cells. Associative memory cells encode new sensory signals alongside innate signals, which are called primary associative memory cells (pAMC). They receive synapse innervations from co-activated sensory cortices alongside innate sensory inputs. Their axons project to the brain areas related to cognition (prefrontal cortex and hippocampus), emotions (amygdala and nucleus accumbens) and behaviors (motor cortex). The downstream neurons can be mutually innervated to encode dual associative signals (secondary associative memory cells, sAMC) during active thinking. sAMC also include those neurons that receive convergence inputs from different groups of pAMC. The mutual innervations can be induced among the different groups of pAMC by the associations of their correspondent intramodal signals. The different groups of associative memory cells and their inputs/outputs are presented by the different intensities of colors, such as light blue *vs* dark blue and orange *vs* red.

Memory to associated signals is primarily fulfilled by associative memory cells in sensory cortices. These associative memory cells recruited from sensory cortical neurons possess the following characteristics ( Figure 1). They encode associated signals, including their innate signals and new signals. They receive new synapse innervations from the co-activated sensory cortices besides their innate sensory input for the integration and storage of associated innate and new signals. Their axons project to brain areas that control behavior, cognition and emotion to initiate memory presentations. Their recruitment is influenced by epigenetics-regulated genes and proteins that are related to memory. In the integration, storage and retrieval of these associated signals, the working principles for associative memory cells are based on their receptions to innate and new synapse inputs for signal integrations, their abilities to convert synaptic analogue signals into digital spikes for encoding the associated signals, and their capacities of spike outputs to drive behavior-, cognition- and emotion-related brain areas. Therefore, the synapse inputs onto associative memory cells determine the specificity of memory contents. The number, activity level and plasticity of associative memory cells, as well as the connection and activity strengths in their input and output partners, set up the power and persistence of information storage and memory presentation. With associative memory cells in the sensory cortices ^1, 3, 7, 16^, their axon-innervated downstream brain cells are able to encode these associated signals ^17– 20^. The stimulations to any of these areas in the neural circuits from sensory cortices to behavior- and emotion-controlled brain nuclei can induce memory presentation ^21– 27^.

## The impact of associative memory cells on physiology and pathology

One signal induces the recall of its associated signals and the expression of their respective behaviors, or the other way around, such that individuals are able to fulfill logical reasoning and associative thinking in forward and backward manners. Each of the co-activated sensory cortices encodes the associated innate signal and newly learnt signal. Each of the associated signals is stored at multiple sensory cortices, and this storage prevents memory loss ^3^. The storage of multiple signals in an associative memory cell strengthens the efficiency of memory retrieval. In addition, the storage of multiple signals in a cortical area and the recall of one signal triggered by multiple signals enable these individuals to strengthen their abilities in memory retrieval and well-organized cognitions.

There are two forms of neuronal plasticity, i.e., the recruitment of associative memory cells, as well as the structural and functional changes of neurons/synapses. The recruitment of associative memory cells driven by new synapse innervations is related to store the specifically acquired signals, which differs from plasticity at the existing synapses, such as potentiation and depression that remain to be proved specifically for the newly acquired signals ^7^. Structure and functional plasticity at the subcellular compartments of associative memory cells determines whether they sensitively integrate associated signals, precisely encode memorized signals and efficiently trigger the neurons in their downstream brain areas for memory presentation ^7^.

A working diagram of associative memory cells in cross-modal memory and reflexes is provided in Figure 1. In addition to the innate input, the activity strength of associative memory cells in the given sensory cortex is facilitated by the newly innervated synapses from other co-activated sensory cortices. For instance, piriform cortical neurons receive synapse innervations from the barrel cortex after the pairings of whisker and odor signals. On the basis of odor signals from the olfactory afferent pathway, the activity of synapse inputs from barrel cortical neurons by whisker stimulus will drive these piriform cortical neurons toward a threshold for firing spikes, and their spikes activate the downstream neurons for olfactory responses, such that a whisker-induced olfactory response is formed. On the other hand, barrel cortical neurons receive synapse innervations from the piriform cortex after the pairings of whisker and odor signals. On the basis of whisker signals from the whisker afferent pathway, the activity of synapse inputs from piriform cortical neurons by odor stimulation will drive these barrel cortical neurons toward a threshold for firing spikes. Their spikes activate the motor cortical neurons for whisker motions, such that odorant-induced whisker motion is formed. In the meantime, the activation of their downstream cognition- and emotion-brain regions will lead to the accompanying responses in emotion and cognition ^7^.

In terms of memory enhancement, weakness and loss, the changes in associative memory cells from the sensory cortices to cognition-, behavior- and emotion-related brain regions are critical. For instance, if the innervations from multi-signal inputs to associative memory cells and their upregulation are persistent in sensory cortices, memory traces will be maintained over a life time. The decayed plasticity at behavior-related cortices, due to the lack of use, may lead to the inability of memory presentation, i.e., memory weakness or loss ^15^. In fact, although certain learned signals cannot be intrinsically and spontaneously recalled, these stored signals can be retrieved from the sensory cortex by the signal, similar to or same as, them. On the other hand, if signals’ integrations at associative memory cells are unusually strong, the hyperactivity of associative memory cells may lead to seizure activities in motor cortices for epilepsy, in sensory cortices for hallucination and in cognitive cortices for delusion ^7^.

## Plasticity at associative memory cells

In glutamatergic neurons, the excitatory synaptic inputs, intrinsic property and axon outputs are upregulated. In GABAergic neurons, the excitatory inputs are upregulated, whereas the intrinsic property and axon outputs are downregulated. These factors may coordinately facilitate the driving force from new synapse innervations to recruit glutamatergic and GABAergic neurons as associative memory cells, and promote their functioning state to store signals ^7^. For instance, in neurons with newly formed synapse innervations, the increased excitatory inputs and decreased inhibitory inputs can increase their active states to a higher level for receiving and storing new information, i.e., the recruitment of associative memory cells ^1, 3, 7^. The increased number and function of excitatory synapse inputs can strengthen the encoding capacity and precision of associative memory cells for information storage and retrieval precisely and efficiently ^28– 30^. If the excitatory associative memory cells are overly active, they may activate the neighboring inhibitory neurons and prevent associative memory cells from hyperactivity via recurrent negative feedback ^28, 31– 33^.

## Perspectives of associative memory cells

In addition to recruitment of associative memory cells by the association of exogenous signals, associative memory cells may be recruited by the association of endogenous signals in the brain during cognition, such as associative thinking and logical reasoning ( Figure 1). In active thinking, the associations of previously stored associative signals in sensory cortices may lead to recruited associative memory cells (i.e., primary associative memory cell) making mutual synapse innervations among them and convergent innervating downstream neurons. In their downstream brain areas, the neurons begin to encode dual associative signals and are recruited to be new associative memory cells (i.e., secondary associative memory cells). The contents of associative thinking and logical reasoning are memorized. In subsequent cognitive activity, the secondary associative memory cells can be activated for mixed associative memory presentation, high-level cognition and even inspiration. Thus, the more associative thinking is, the higher the integration of associative memory cells and the more inspiration there is ^3^. Moreover, secondary associative memory cells may be recruited by giving exogeneous associated signals to pair two forms of associative memory. In terms of the connections among different groups of associative memory cells, the patterns of sequential links and a common-shared group may be present for signals’ integrations.

In addition to associative learning through cross-modal sensory systems to induce cross-modal associative memory, associative learning can be intramodal, e.g., associated images to the visual system, associated odors to the olfactory system, associated words to the auditory system, associated somatosensory signals to the somatosensory system, and so on, inducing intramodal associative memory. The associated signals from a given sensory input to its sensory cortex may initiate two sets of neurons that encode each of these associated signals to form mutual innervation through axons and synapses, so that associative memory cells in a single modality of the sensory cortex are recruited. In this given sensory cortex, associative memory cells are formed to memorize intramodal signals with the different features, strengths and locations of input signals ( Figure 1). With the associative memory cells in intramodal sensory cortices, intramodal memory to associated signals is formed, e.g. image one induces image two recall, odor one induces odor two recall and word one induces word two recall, or the other way around. It is noteworthy that there is a time delay among intramodal signals, in which the activity persistence in sets one and two of neurons in this given sensory cortex controls whether their co-activations overlap to recruit intramodal associative memory cells. The different portions, activity strengths and connections of these neurons are responsible for the storage and retrieval of intramodal signals with different features ^34^.

 During associative thinking and logical reasoning, we usually can tell that images are from previous sights, words from previous reading or listening, tastes from previous eating, and so on. These phenomena indicate that the recalled signals originate from associative memory cells in sensory cortices, and/or that the secondary associative memory cells in cognitive cortices send synapse innervations back to the primary associative memory cells. Their interactions make associative thinking and logical reasoning with the inclusions of the sensory origins. In addition, images, odors, tastes and events are presented by the word-based language during associative thinking and logical reasoning. In initial learning, these sensations/events and their word descriptions are associated, such that associative memory cells for encoding these sensations/events and word descriptions have been recruited. Once sensations and behaviors are recalled in sequential playbacks, their word descriptions in these associative memory cells are initiated to substitute the complicated images and events for speeding up these cognitive processes. However, if words and sensation/events are associated improperly, the correction of these associations is difficult because of the presence of these recruited synapse innervations, associative memory cells and their circuits.

The formation of associative memory cells in terms of number and distributed areas is affected by the excitatory state of the brain. More excitatory strengths and areas recruit more associative memory cells, i.e., activity together and connection together. When the brain is highly excited in many areas, such as euphoria perception, extreme fear and strong stimulus, more associative memory cells are recruited in these areas through their mutual innervations, so that impressive memory and spontaneous recall to these experiences are generated in an individual’s life ^7^. In this regard, it is difficult to remove these formed synapse innervations and recruited associative memory cells for the relief of fear memory. Alternative ways can be the avoidance of fear stimulations and the induction of happiness to rebalance these two states toward the weakness of fear memory, since the lack of uses in neural circuits related to fear memory may drive them to be function silence. In the brains of individuals with a history of substance abuse or addiction, associative memory cells are formed to be large in number and are found in extensive areas, meaning that relapses occur during the individual’s life time ^1^. Strategies for these individuals may include the avoidance of the environmental cues associated with substance abuse to silence these associative memory cells and circuits, as well as establish alternative happiness to modify these silent associative memory cells.

 How different groups of associative memory cells work together during dreaming is proposed below. Dreams are usually associated with high activities in electronic encephalograph and other behaviors, such as rapid eye movement, muscle twitch and active respiration/heat beat, indicating high activity in the cerebral brain. In the meantime, associative memory cells are presumably activated, especially those for images and events intensively activated and frequently thought in daytime, such that the images and events in more or less similarity are playbacks. In other words, associative thinking and logical reasoning based on associative memory cells can be fulfilled under an unawake condition. The incompletely identical images and events in the dreams may be caused by the differential integration of associative memory cells when the brain is not fully awake, compared to the awake condition ^7^.

In terms of molecular mechanisms underlying the recruitment of associative memory cells, epigenetic-mediated processes are presumably involved ^6, 7, 15^, since their formations are triggered by the external inputs from sensory organs and the intrinsic activation by endogenous synapse inputs. The downstream molecules and signaling pathways of these epigenetic events that regulate synapse formation and neuron/synapse activities are presumably contributing to the recruitment of associative memory cells, which remains to be tested.

**Figure 2.**
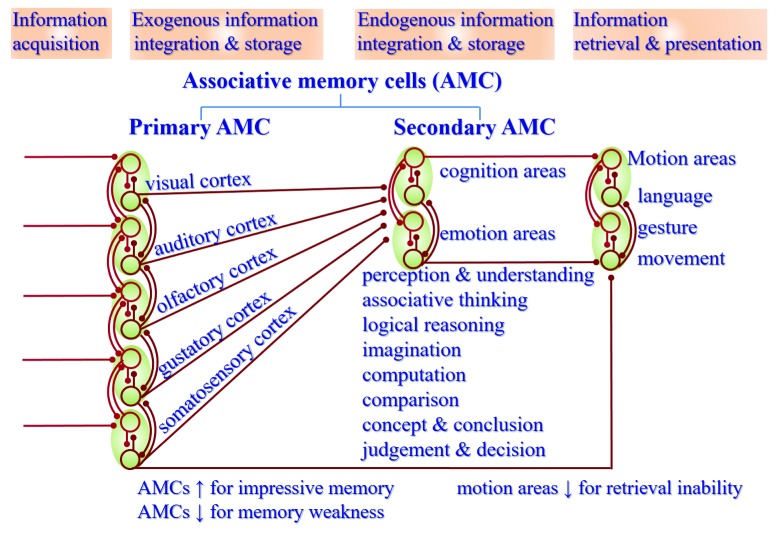
Associative memory cells and their roles in the memory, cognition and emotion. Associative learning and memory can be divided into four stages, i.e., information acquisition, exogenous information integration and storage, endogenous information integration and storage as well as information retrieval and presentation. Based on the sources of information integration and storage, associative memory cells (AMC) are classified into primary AMCs in sensory cortices to memorize exogenous information and secondary AMCs in cognition-, emotion- and behavior-related brain areas to memorize endogenous information during associated cognition and emotion events. Cross-modal associative memory cells are recruited by mutual innervations among sensory cortices or between cognition- and emotion-related brain areas. Intramodal associative memory cells are recruited by mutual innervations among neurons in a single-modality sensory cortex, cognition brain area or emotion brain area. The upregulations of the AMC number and active strength facilitate memory to be impressive, or vice versa. The functional downregulation of motion-related brain areas leads to the inability of memory retrieval and presentation.

Taken these studies together, the classification of associative memory cells is given in Figure 2. Based on their roles in the integration and storage of information sources, associative memory cells in the sensory cortices that are formed after initial associative learning to memorize exogenous information are named as primary associative memory cells, and those memory cells in the cognition-, emotion- and behavior-related brain areas recruited during associated cognition and emotion events to memorize endogenous information are named as secondary associative memory cells. Primary associative memory cells include cross-modal associative memory cells recruited by mutual innervation among sensory cortices and intramodal associative memory cells recruited by mutual innervation among neurons in single-modality sensory cortex during their co-activations. For instance, associative memory cells for the whisker and odor signals (woAMC) and for the words and events (weAMC) are cross-modal in nature. Similarly, secondary associative memory cells can be cross-modal between cognition and emotion or between sensation and cognition/emotion, as well as intramodal in cognitions or emotions.

## Conclusions

In summary, based on studies of associative memory cells, we have constructed a working map of the brain for the integration, storage and retrieval of associated signals, as well as the subsequent cognitive processes. Associative memory cells and their activity-dependent activation play important roles in associative memory and cognition. The perspectives in this review are expected to be useful for future study to scheme comprehensive brain-working atlases.
