# Supplementary material for: Associative memory cells: Formation, function and perspective
Source: F1000Res. 2017 Mar 30;6:283. Originally published 2017 Mar 17. [Version 2] doi: 10.12688/f1000research.11096.2 (PMC5373424; doi:10.12688/f1000research.11096.2)
Supplement: Peer review history

# Peer review: Referee response for version 2

**DOI:** 10.5256/f1000research.12198.r21364

**Published:** 2017-03-31

**Authors:** Hongxin Dong

**Affiliations:** 1 Department of Psychiatry and Behavioral Sciences, Feinberg School of Medicine, Northwestern University , Chicago, IL, USA

I have read this submission. I believe that I have an appropriate level of expertise to confirm that it is of an acceptable scientific standard.

# Peer review: Referee response for version 1

**DOI:** 10.5256/f1000research.11969.r21289

**Published:** 2017-03-27

**Authors:** Hongxin Dong

**Affiliations:** 1 Department of Psychiatry and Behavioral Sciences, Feinberg School of Medicine, Northwestern University , Chicago, IL, USA

In this review Dr. Jin-Hui Wang and Shan Cui proposed a model named “associative memory cells (AMCs)” that could be used to explain associative memory formation and functioning at the neuronal circuitry level. This novel cellular model was first established in Dr. Wang’s laboratory. Based on their serial studies, they proposed the hypothesis that AMCs encode both new and innate signals based on their mutual synapse innervation in the sensory cortical area. Furthermore, they suggested that the primary associative memory cells innervate the downstream neurons in associated brain areas, and the downstream neurons encode dual associative signals and serve as the secondary associative memory cells (sAMCs). The formation and functioning of the AMCs are very interesting and important as they may help to reveal novel mechanisms for learning, memory and cognition.

I have read this submission. I believe that I have an appropriate level of expertise to confirm that it is of an acceptable scientific standard.

# Peer review: Referee response for version 1

**DOI:** 10.5256/f1000research.11969.r21064

**Published:** 2017-03-27

**Authors:** Jian-Guo Chen

**Affiliations:** 1 Laboratory of Neuropsychiatric Diseases, Institute of Brain Research, Collaborative Innovation Center for Brain Science, Huazhong University of Science and Technology (HUST), Wuhan, China

This review summarizes current studies about cellular mechanisms underlying associative memory, in which associative memory cells are recruited for the integration, storage and retrieval of cross-modal signals. The contents in this review are and advanced concept. To improve this review, I would suggest that authors include the following information.

 1) The physiological and pathological impacts are caused by the quantity of associative memory cells.

 2) How many kinds of associative memory cells are present in the cerebral cortices?

I have read this submission. I believe that I have an appropriate level of expertise to confirm that it is of an acceptable scientific standard.

# Peer review: Referee response for version 1

**DOI:** 10.5256/f1000research.11969.r21274

**Published:** 2017-03-27

**Authors:** Ping Zheng

**Affiliations:** 1 State Key Laboratory of Medical Neurobiology, School of Basic Medical Sciences, Institutes of Brain Science (IOBS), Fudan University, Shanghai, China

Associative learning and memory is a very interesting topic. Jin-Hui Wang *et al. *summarize associative memory cells in their formation, features and functional impacts. The review is well finished. I have the following suggestions:

 1. P1: The definition for associative learning: ”Associative learning is a common approach for information acquisition” is a bit too general. I suggest that using “Associative learning is the learning of associations between events” to replace it.

 2. Give a definition for “associative memory cells” and provide evidence to support “associative memory cells”.

 3. P2: Since “In the studies of cellular and molecular mechanisms underlying associative learning and memory, animal models in fear conditioning, eyelid-blinking conditioning and operant conditioning are used”, the following description for associative memory cells should be advanced using these models. The present description for associative memory cells is a bit too general.  

I have read this submission. I believe that I have an appropriate level of expertise to confirm that it is of an acceptable scientific standard.
